# ^99m^Tc-Labeled Cyclic Peptide Targeting PD-L1 as a Novel Nuclear Imaging Probe

**DOI:** 10.3390/pharmaceutics15122662

**Published:** 2023-11-23

**Authors:** Guillermina Ferro-Flores, Blanca Ocampo-García, Pedro Cruz-Nova, Myrna Luna-Gutiérrez, Gerardo Bravo-Villegas, Erika Azorín-Vega, Nallely Jiménez-Mancilla, Emiliano Michel-Sánchez, Osvaldo García-Pérez, Nancy Lara-Almazán, Clara Santos-Cuevas

**Affiliations:** 1 Department of Radioactive Materials, Instituto Nacional de Investigaciones Nucleares, Ocoyoacac 52750, Mexico; 2 Faculty of Chemistry, Universidad Autónoma del Estado de México, Toluca 50180, Mexico; 3 Cátedras, CONACyT, Instituto Nacional de Investigaciones Nucleares, Ocoyoacac 52750, Mexico; 4 Department of Nuclear Medicine, Instituto Nacional de Cancerología, Tlalpan, Mexico City 14080, Mexico

**Keywords:** PD-L1, technetium-99m, PD-L1 peptide inhibitor, SPECT, PD-L1 imaging

## Abstract

Recent cancer therapies have focused on reducing immune suppression in the tumor microenvironment to prevent cancer progression and metastasis. PD-1 is a checkpoint protein that stops the immune response and is expressed on immune T cells. Cancer cells express a PD-1 ligand (PD-L1) to bind to the T-cell surface and activate immunosuppressive pathways. This study aimed to design, synthesize, and evaluate a ^99m^Tc-labeled PD-L1-targeting cyclic peptide inhibitor (^99m^Tc-iPD-L1) as a novel SPECT radiopharmaceutical for PD-L1 expression imaging. AutoDock software (version 1.5) was used to perform molecular docking for affinity calculations. The chemical synthesis was based on the coupling reaction of 6-hydrazinylpyridine-3-carboxylic acid with a 14-amino-acid cyclic peptide. iPD-L1 was prepared for ^99m^Tc labeling. Radio-HPLC was used to verify radiochemical purity. The stability of the radiopeptide in human serum was evaluated by HPLC. iPD-L1 specificity was assessed by SDS-PAGE. [^99m^Tc]Tc-iPD-L1 cellular uptake in PD-L1-positive cancer cells (HCC827 and HCT116) and biodistribution in mice with induced tumors were also performed. One patient with advanced plantar malignant melanoma received [^99m^Tc]Tc-iPD-L1. The iPD-L1 ligand (AutoDock affinity: −6.7 kcal/mol), characterized by UPLC mass, FT-IR, and UV–Vis spectroscopy, was obtained with a chemical purity of 97%. The [^99m^Tc]Tc-iPD-L1 was prepared with a radiochemical purity of >90%. In vitro and in vivo analyses demonstrated [^99m^Tc]Tc-iPD-L1 stability (>90% at 24 h) in human serum, specific recognition for PD-L1, high uptake by the tumor (6.98 ± 0.89% ID/g at 1 h), and rapid hepatobiliary and kidney elimination. [^99m^Tc]Tc-iPD-L1 successfully detected PD-L1-positive lesions in a patient with plantar malignant melanoma. The results obtained in this study warrant further dosimetric and clinical studies to determine the sensitivity and specificity of [^99m^Tc]Tc-iPD-L1/SPECT for PD-L1 expression imaging.

## 1. Introduction

Immune checkpoint inhibitor (ICI) therapy has become an important therapeutic option for treating various cancers [1,2,3,4,5]. Programmed death receptor 1 (PD-1) and the PD-L1 ligand (PD-L1) are the major molecular targets for ICI therapy among the targeted immune checkpoints. PD-L1 has been shown to be a repeatable marker. It can be used to guide therapeutic decisions and monitor response [1]. However, PD-L1 expression is not only heterogeneous between and within tumor lesions but also highly dynamic, changing over time. Therefore, the detection of PD-L1 by immunohistochemical techniques needs to be complemented by other diagnostic modalities. On the other hand, molecular imaging techniques such as single photon emission tomography (SPECT) and positron emission tomography (PET) have the advantage of non-invasive, in vivo, whole-body scanning with high sensitivity, adequate spatial resolution, and image acquisition times in minutes. Thus, they could visualize the heterogeneous PD-L1 gene expression between tumor lesions in patients over time [6].

The preclinical development of PD-L1 inhibitory radiopharmaceuticals for SPECT and PET is based on three different types of molecules: antibodies, peptides, and small non-peptide molecules [6]. Of these, only antibodies and peptides have been used in humans for PET imaging [7,8] and none for SPECT imaging. High tissue accumulation has been observed for a variety of radiolabeled antibody types (minibodies, affibodies, and nanobodies). However, these molecules have a relatively high degree of immunogenicity and can even induce potentially adverse immunological effects such as cytokine storms. In contrast, peptide and non-peptide small molecules accumulate more rapidly in tumor tissue, allowing same-day patient imaging. The release of the radiopharmaceuticals is fast and takes place within the favorable time window of a few minutes to a few hours. They also have the advantage of being manufactured under Good Manufacturing Practice (GMP) protocols. In the last few years, a number of important small molecule and peptide-based inhibitors have been developed for PD-1/PD-L1 blockade [9,10,11]. In addition, small molecules have the advantage of high tissue penetration and cell membrane permeability to reach defined molecular targets [12,13,14,15].

**Figure 1:**
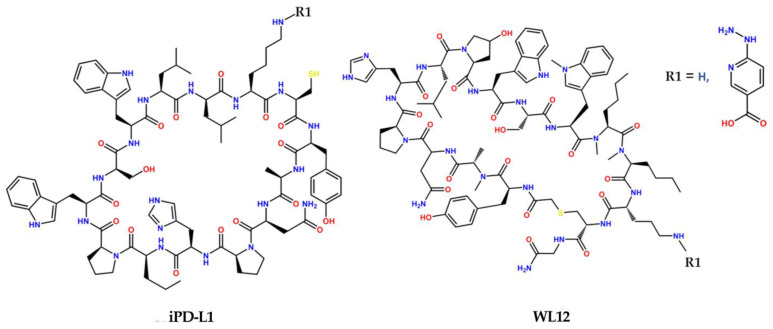
Schematic structures of the programmed death ligand inhibitor peptides named iPD-L1 (designed and evaluated in this research; R1 = H) and WL12 (positive control; R1 = H) and their HYNIC derivatives.

In 2002, the first human PET imaging of PD-L1 expression in lung cancer patients was published with excellent uptake and specificity [8]. The peptide used was a cyclo-peptide (14 amino acids) named WL12 with four methylated amino acids (Trp(Me), NMeAla, and two NMeNle) and a ^68^Ga-NOTA-conjugated side chain of the Orn residue for PET imaging. We hypothesized that the use of the HYNIC (pyridine-hydrazine) heterocycle would provide the appropriate anchorage to the hydrophobic sites of the target protein [13,14,15], allowing the elimination of the four methylation sites and the removal of the -S- bond from the cyclic amino acid chain to obtain a more rigid structure. In addition, Orn could be replaced by Lys to obtain a longer side chain that does not interfere with the recognition site, thus improving the affinity of the PD-L1 inhibitory peptide (iPD-L1) upon ^99m^Tc-HYNIC conjugation (SPECT image) (Figure 1).

The aim of this study was to design, synthesize, and evaluate a ^99m^Tc-labeled cyclic HYNIC-peptide inhibitor ([^99m^Tc]Tc-iPD-L1) as a new SPECT radiopharmaceutical for PD-L1 expression imaging.

## 2. Materials and Methods

### 2.1. Docking 

The ligands iPD-L1 and HYNIC-iPD-L1, as well as WL12 (positive control) and HYNIC-WL12 (positive control), were modeled in two dimensions (2D) using ChemDraw to obtain the basic structures. The three-dimensional structure of each ligand was exported in mol2 format using Chem3D to optimize the molecular geometry using the MMFF94 force field via the OpenBabel chemical toolbox. PD-1 and PD-L1 obtained from the protein database (PDB ID: 4ZQK) were used as the receptor, which was edited to remove water molecules, ions, and structures foreign to PD-L1 from the X-ray crystallography process. For virtual screening, each of the structures shown in Figure 1 was used as a ligand.

The receptor as a macromolecule and the corresponding ligands were prepared using the AutoDock Tools 1.5.7 graphics package, with the search box set cubically 80 Å on the x, y, and z axes, centered in the macromolecule. Semi-rigid docking was performed with AutoDock vina 1.1.2 around the entire receptor surface, generating 20 poses for each ligand. Inhibition constants were calculated as previously reported [13]. 

### 2.2. Synthesis and Chemical Characterization 

Succinimidyl 6-Boc-hydrazinopyridine-3-carboxylate (NHS-HYNIC-Boc) was purchased from Synchem UG & Co (Felsberg, Germany). The cyclo(Trp-Ser-Trp-Leu-Leu-Lys-Cys-Tyr-Ala-Asn-Pro-His-Leu-Pro) (iPD-L1) and cyclo(AcTyr-MeAla-Asn-Pro-His-Leu-Hyp-Trp-Ser-Trp(Me)-MeNle-MeNle-Orn-Cys)-Gly-NH_2_ (thioether bridge: Cys14-Ac-Tyr) (WL12; positive control) peptides were custom synthesized for our laboratory by Shanghai Yaxian Chemical Co, Ltd. (Jiading, Shanghai, China). 

The iPD-L1 (6 mg; 3.5 µmol) or WL12 (6 mg; 3.2 µmol) peptide was dissolved in 0.5 mL dimethylformamide (DMF), followed by the addition of N,N-diisopropylethylamine (20 µL). NHS-HYNIC-Boc (2 mg; 5.7 µmol) was dissolved in 100 µL DMF and added to the peptide solution for 24 h at room temperature. Unreacted NHS-HYNIC-Boc was removed from the reaction mixture by dialysis (Tube-O-DIALYZER™ mini dialysis system, 1 kDa MWCO; Merck; Burlington, MA, USA) for 24 h using injectable-grade water. To the resulting cloudy aqueous solution (0.5 mL), 1 mL trifluoroacetic acid (TFA) was added to deprotect the HYNIC (Boc removal), and the mixture was kept at room temperature for 2 h. The dialysis process was then repeated. Acetonitrile was added to the cloudy solution obtained in a final ratio of 40:60 acetonitrile: water to completely dissolve the conjugate. Finally, the conjugate was purified by HPLC (Discovery^®^ C18 HPLC column 5 μm particle size, L × I.D. 25 cm × 10 mm) (Merck; Burlington, MA, USA) using a linear gradient of 0.1% TFA-water/0.1% TFA-acetonitrile from 100 to 20% of the aqueous phase in 20 min at a flow rate of 4 mL/min. The fraction collected between 13.5 and 15 min was lyophilized. 

The resulting powder was analyzed by IR-FT vibrational spectroscopy (400–4000 cm^−1^, 50 scans at 0.4 cm^−1^; FT-IR 660 spectrometer, Agilent Technologies). The HYNIC peptide dissolved in ethanol: water 40:60 was used for characterization by UPLC mass spectroscopy (ADQUITY UPLC H-Class with QDa mass detector; Waters Corporation, Milford, CT, USA) and UV–vis spectroscopy in the range of 200–400 nm (PerkinElmer LambdaBio spectrometer; Waltham, MA, USA).

### 2.3. Radiolabeling 

HYNIC-WL12 and HYNIC-iPD-L1 peptides were dissolved in a 1:1 ethanol: water solution at a concentration of 1 mg/mL. For ^99m^Tc labeling, 100 µL of peptide solution, 500 µL tricine (N-[tris(hydroxymethyl) methyl]glycine)/EDDA (ethylenediamine-N,N′-diacetic acid) solution (60 mg tricine in 1.5 mL 0.2 M phosphate buffer at pH 7/30 mg EDDA in 1.5 mL 0.1 M NaOH) and 500 µL ^99m^TcO_4_Na (GETEC, ININ, Ocoyoacac, Mexico) with an activity of 1110 MBq were mixed, followed by 20 µL SnCl_2_ (10 mg/10 µL HCl conc. in 10 mL water). The solution was stirred for 1 min, then incubated in a dry bath at 95 °C for 30 min and allowed to cool to room temperature for 10 min. A batch of the lyophilized formulation containing EDDA, tricine, SnCl_2_, and mannitol was also prepared in an aseptic area under good manufacturing practices (GMP-certified area) for reconstitution with sodium pertechnetate-99m solution as previously reported [13,14,15].

### 2.4. Quality Control 

For quality control of the [^99m^Tc]Tc-iPD-L1 and [^99m^Tc]Tc-WL12 formulations, parameters such as appearance (clear solution), pH (neutral), sterility, absence of bacterial endotoxins, and radiochemical purity (radio-HPLC; Waters Corporation, Milford, CT, USA; 3.9 mm × 30 cm µBondapak™ C18 column; linear gradient system; solvent A: 0.1% TFA-acetonitrile and solvent B: 0.1% TFA-water, from 100 to 30% of A in 20 min) were evaluated according to the Mexican Pharmacopoeia [16], in its section on “General Methods of Analysis” (MGA). The retention time of [^99m^Tc]Tc-WL12 was 15.0 ± 0.3 min and 15.5 ± 0.2 min for [^99m^Tc]Tc-iPD-L1, while the retention times of ^99m^TcO_4^−^_ and [^99m^Tc]Tc-EDDA/tricine were 3.5 ± 0.2 min and 4.5 ± 0.2 min, respectively. 

### 2.5. Stability 

The stability of [^99m^Tc]Tc-iPD-L1 and [^99m^Tc]Tc-WL12 was evaluated by diluting the samples in human serum (5x). The radiopharmaceuticals (n = 3) were heated at 37 °C and analyzed at 0.5, 3, and 24 h for radiochemical purity by size-exclusion radio-HPLC (ProteinPak 300SW, Waters Corporation, Milford, CT, USA).

### 2.6. Molecular Recognition by the PD-L1 Protein: Radio-SDS-PAGE 

SDS-PAGE gel electrophoresis was performed. Radioactivity distribution was determined using a radio-TLC scanner (Mini-Gita, 60–150 keV, BGO-Crystal, window 25 *×* 2 mm, thickness 5 mm, resolution optimized for Tc-99m; RayTest, Munich, Germany) to detect [^99m^Tc]Tc-iPD-L1 and [^99m^Tc]Tc-WL12 binding to PD-L1 protein. 

Briefly, ^99m^Tc-labeled WL12 and iPD-L1 solutions (100 µL) (1 mg/mL peptide) were incubated with human recombinant PD-L1 protein (100 µL) (200 µg/mL; FineTest; Cat: P6857) (radiopeptide-PD-L1 interaction) or human integrin αVβ3 protein (100 µL) (negative control; 200 µg/mL) (Chemicom, Merck; Burlington, MA, USA) (radiopeptide-integrin interaction) for 30 min at 37 °C, after which the samples were diluted with Laemmli sample buffer (containing 10% SDS, Tris-Cl 125 mM, pH = 6.8, 12.5% glycerol and 0.005% bromophenol blue after dye) to give final protein concentrations of 25 µg/mL. Samples (^99m^Tc-WL12, ^99m^Tc iPD-L1, PD-L1 protein, integrin, ^99m^Tc-WL12-PD-L1 protein, ^99m^Tc iPD-L1-PD-L1 protein, ^99m^Tc-WL12-integrin, and ^99m^Tc iPD-L1-integrin) were loaded onto the SDS-PAGE gel at room temperature. Thirty µL (30 µg) of each sample was loaded into 1.5 mm thick wells of 15% polyacrylamide gels. The experiments were performed on a single gel. In all cases, electrophoresis was run until just before the bromophenol dye reached the bottom of the gel (total gel distance of 100 mm). Pre-set molecular weight standards (BLUEstainTM Protein Ladder 11–245 kDa Cat: P007-500) were also run on each gel. The gels were stained with Coomassie Blue for 30 to 45 min at room temperature and allowed to fade for 4 to 20 h before photography. Finally, the radioactivity distribution on the gels was scanned using a radio-TLC detector to determine the percentage of [^99m^Tc]Tc-iPD-L1 or [^99m^Tc]Tc-WL12 radioactivity associated with proteins.

### 2.7. Immunofluorescence

Human colorectal cancer HCT116 (PD-L1 positive: 45.1 nTPM), human lung cancer HCC827 (PD-L1 positive: 27.1 nTPM), and mouse C6 glioma cells (PD-L1 negative control) were obtained from ATCC (Atlanta, GA, USA). FBS, RPMI, and cell culture medium were obtained from Bovine and Biology (CDMX, MX). Cells were cultured in RPMI medium (5% CO_2_; 100 µg/mL penicillin, 15% fetal bovine serum, and 100 µg/mL streptomycin; 37 °C).

Immunofluorescence was used to confirm cellular PD-L1 expression. HCC827, HCT116, and C6 cells were fixed with 4% paraformaldehyde for 20 min. Cells were treated with 0.5% Triton X-100 for permeabilization and blocked with 1% BSA. Cells were then incubated overnight with an anti-PD-L1 antibody according to the supplier’s instructions (FineTest, Wuhan Fine Biotech Co., Wuhan, China). After incubation, the fluorescence intensity of the cells was observed with Alexa Fluor488-conjugated goat anti-mouse IgG (H + L) (Invitrogen cat. no. A32731). Microscopic observation of fluorescence was performed (Meiji Techno; model MT-6200; Saitama, Japan).

### 2.8. Cellular Uptake and Internalization

Cells diluted in phosphate-buffered saline (PBS) (pH 7.4) (1 × 10^6^ cells/tube) received three different treatments: (a) [^99m^Tc]Tc-iPD-L1 (7.4 kBq) (n = 3), (b) and [^99m^Tc]Tc-WL12 (7.4 kBq) (n = 3), and (c) ^99m^TcO_4_Na (7.4 kBq) (n = 3). Cells were incubated with each treatment at 37 °C for one hour. After incubation, tubes were measured in a NaI(Tl) detector (NML Inc., Houston, TX, USA) to establish the initial or first activity (100%). The tubes were spun at 500 g for 10 min, rinsed once with PBS, and spun once more. The liquid was removed, and the activity in the button was determined, indicating the percentage of cellular uptake (surface uptake) regarding the initial or first activity. Acetic acid/0.5 M NaCl was added, the tubes were centrifuged, and the activity was measured. The liquid was removed, and activity in button activity represented the percentage of activity internalized by cells relative to uptake activity (internalization fraction of surface uptake).

### 2.9. Biodistribution 

All animal procedures were performed following the Institutional Animal Care and Use Committee requirements under an approved protocol (No. 09-2018-2022) and the Ethical Regulations for the Handling of Laboratory Animals (NOM-062-ZOO-1999). Male Nu/Nu (CINVESTAV, I.PN., Mexico City, Mexico) mice, 6–8 weeks of age, were maintained in an aseptic barrier environment. Mice were inoculated by subcutaneous injection of 1 × 10^6^ HCC827 cells/0.1 mL PBS into the upper back. Injection sites were monitored periodically for tumor progression. On day ten after tumor cell inoculation, animals were injected with 18.5 MBq (50 µL) of [^99m^Tc]Tc-iPD-L1 or 18.5 MBq (50 µL) of [^99m^Tc]Tc-WL12 by tail vein injection. The animals were sacrificed at 1, 3, and 24 h (n = 3). Kidneys, lungs, heart, liver, spleen, pancreas, small intestine, stomach, muscle, and blood and tumor samples were dissected. Radioactivity was determined in a sodium iodide detector. The results were stated as % injected dose/g. 

### 2.10. Clinical Imaging 

A 62-year-old man (weight 60 kg; height 168 cm) diagnosed with advanced plantar malignant melanoma was enrolled in this study (Ethics Committee: Protocol No. 2023-MN02). The trial was approved based on the microdose concept and proof-of-concept studies. In addition, the ethical standards of the INCan Hospital based on the Declaration of Helsinki regarding human experimentation and the GMP certificate granted to ININ by the Mexican Ministry of Health were taken into account. The patient signed an informed consent form after receiving detailed information about the purpose of the study, which could help decide on treatment and monitor disease progression. The patient underwent SPECT/CT (Symbia TruePoint, Siemens) 2 h after intravenous administration of [^99m^Tc]Tc-iPD-L1 (740 MBq) and had a previous [^18^F]FDG PET/CT (Excel 20; Siemens Medical Solutions) scan 5 days earlier.

## 3. Results

### 3.1. Docking 

AutoDock software was used to perform molecular docking for affinity calculations. Table 1 shows the scoring results obtained for each inhibitor peptide.

**Figure 2:**
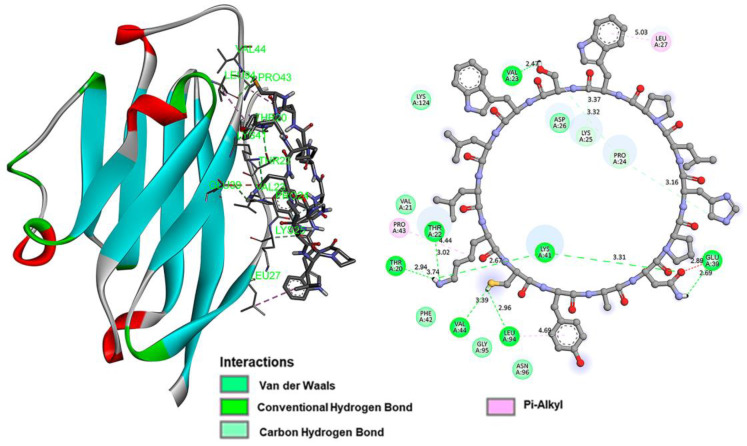
The iPD-L1 ligand has the hydrogen atom as a substituent of the R1 group (Figure 1). The image on the left shows the docking of the iPD-L1 ligand with the PD-L1 protein, interacting with the residues described in Table 1. The right side shows the intermolecular interactions between the ligand and the PD-L1 receptor. The structure in the bar representation corresponds to the ligand, and the pictures in colored spheres represent the amino acids of the PD-L1 receptor that participate in the interaction and the distances are shown in Å scale.

**Figure 3:**
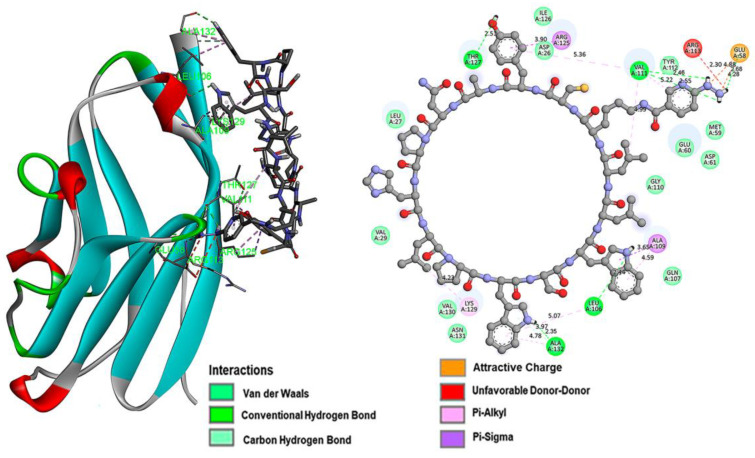
The iPD-L1 ligand has the HYNIC molecule as a substituent of the R1 group (Figure 1). The image on the left shows the docking of the iPD-L1 ligand with the PD-L1 protein, interacting with the residues described in Table 1. The right side shows the intermolecular interactions between the ligand and the PD-L1 receptor, and the distances are shown in Å scale.

**Figure 4:**
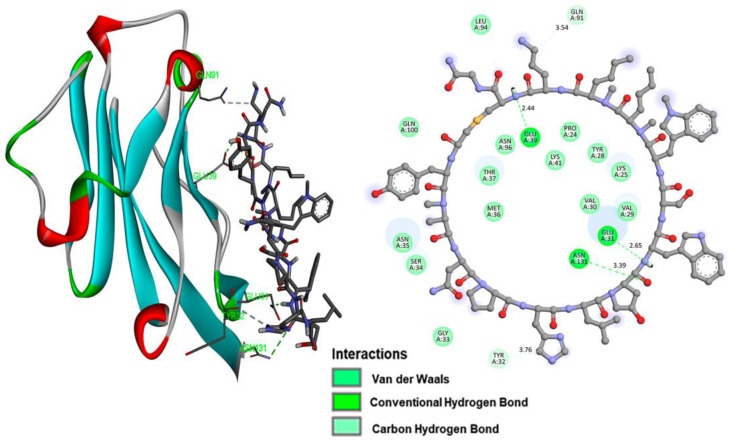
The WL12 ligand has the hydrogen atom as a substituent of the R1 group (Figure 1). The image on the left shows the docking of the WL12 ligand with the PD-L1 protein, interacting with the residues described in Table 1. The right side shows the intermolecular interactions between the ligand and the PD-L1 receptor, and the distances are shown in Å scale.

**Figure 5:**
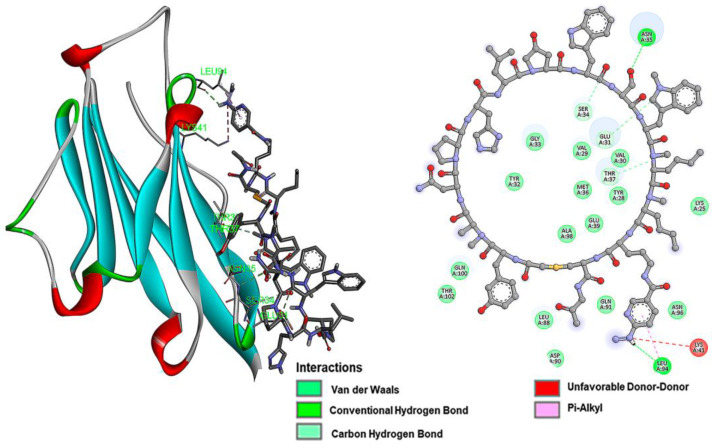
The HYNIC-WL12 ligand has the HYNIC molecule as a substituent of the R1 group (Figure 1). The image on the left shows the docking of the HYNIC-WL12 ligand with the PD-L1 protein, interacting with the residues described in Table 1. The right side shows the intermolecular interactions between the ligand and the PD-L1 receptor.

Intermolecular interaction analysis of the PD-L1 protein complex formed with each of the inhibitory peptides is shown in Figure 2 (iPD-L1), Figure 3 (HYNIC-iPD-L1), Figure 4 (WL12), and Figure 5 (HYNIC-WL12). As expected, the addition of HYNIC as a side chain to the peptide macrocycles of PD-L1 and W12 induces changes in the interaction sites with the PD-L1 protein and, consequently, in the molecular binding energy scoring function (kcal/mol), which is directly related to the affinity. In other words, for both PD-L1 and W12, the presence of HYNIC increases their affinity by decreasing the value of the scoring function linked to the binding potential energy (kcal/mol). Specifically, HYNIC-PD-L1 is the molecule that showed the highest affinity (Table 1), which may be related to the more rigid nature of its cyclic structure due to the absence of the -S- bound (present in WL12), allowing a greater number of interactions with the protein through HYNIC located outside the macrocycle (Figure 3).

### 3.2. Synthesis and IR, Mass, and UV–Vis Spectroscopy Analyses

**Figure 6:**
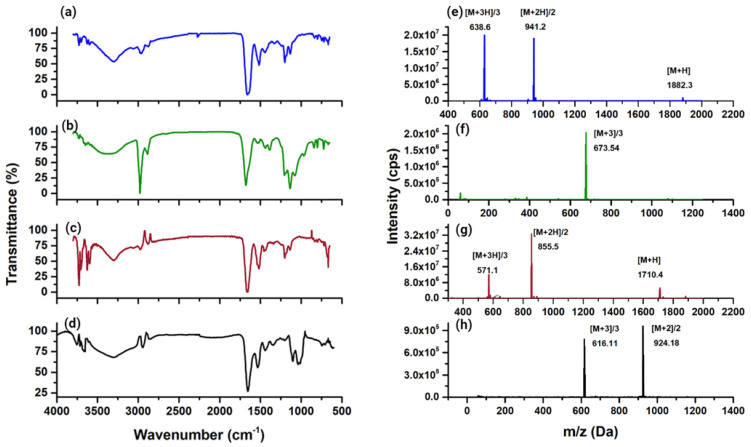
Infrared spectra of (**a**) WL12, (**b**) HYNIC-WL12, (**c**) iPD-L1, and (**d**) HYNIC-iPD-L1 peptides. Mass spectra of (**e**) WL12, (**f**) HYNIC-WL12, (**g**) iPD-L1, and (**h**) HYNIC-iPD-L1 peptides.

The overall chemical yield of the HYNIC conjugates was 60%. In the FT-IR analysis (Figure 6), the aliphatic primary amine band centered at 3295 cm^−1^ from Orn-NH_2_ or Lys-NH_2_ was observed in both WL12 (Figure 6a) and iPD-L1 (Figure 6c) spectra, respectively. After conjugation, the contribution of the primary amine was no longer appreciated and the presence of a new band at 1079 cm^−1^ was shown. In other words, the characteristic C-N stretching vibration of aliphatic secondary amides (at 1079 cm^−1^) is present in both HYNIC-WL12 (Figure 6b) and HYNIC-iPD-L1 (Figure 6d) spectra, confirming the formation of the amide bond between Lys-NH_2_ or Orn-NH_2_ and the carboxyl group of HYNIC. In addition, the N-H vibration of the hydrazine group was observed at 3307 cm^−1^ in the spectra of the HYNIC conjugates (Figure 6c,d). The bands at 2967 and 2877 cm^−1^ correlated with the stretching C-H vibration and 1438 cm^−1^ with the bending C-H vibration, which shifted to 2979 cm^−1^, 2886 cm^−1^, and 1442 cm^−1^, respectively, due to the molecular structural change. The νC-N amines were observed at 1134 cm^−1^ in WL12. In HYNIC-WL12, they were shifted to 1136 cm^−1^. The contribution of the -C=O motif of the amides present in WL12, observed at 1662 cm^−1^ and 1639 cm^−1^, was shifted to 1677 cm^−1^ after HYNIC conjugation (Figure 6c). For iPD-L1, the C-H signals originally at 2981 cm^−1^ and 2883 cm^−1^ were shifted to 2944 cm^−1^ and 2865 cm^−1^ in the HYNIC-iPD-L1 spectrum, and the -C=O vibration of the amides observed at 1662 cm^−1^ and 1639 cm^−1^ was shifted to 1654 cm^−1^ after conjugation (Figure 6d).

The mass spectra of WL12 and iPD-L1 peptides showed the corresponding signals at *m*/*z* 1881 (cal. 1882) [M + H]+ and *m*/*z* 1709 (cal. 1709) [M + H]+, respectively (Figure 6e,g). The respective HYNIC conjugates showed the following signals: HYNIC-WL12 (Figure 6f) with *m*/*z* 2016 (calc. 673) [M + 3]/3 and HYNIC-iPD-L1 (Figure 6h) with *m*/*z* 1845 (calc. 616) [M + 3]/3 and *m*/*z* 1845 (calc. 924) [M + 2]/2.

**Figure 7:**
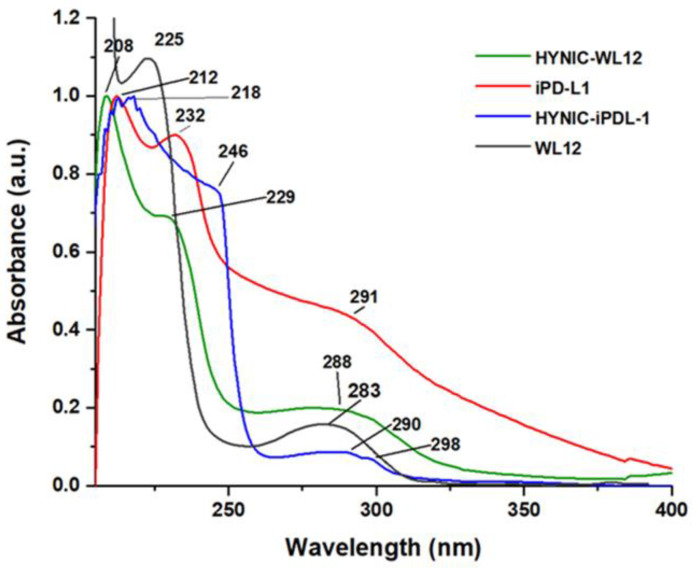
UV–vis spectra of WL12, HYNIC-WL12, iPD-L1, and HYNIC-iPD-L1 peptides.

In the UV–vis spectrum of iPD-L1 (Figure 7), the *n*→σ* contribution to the absorption band from the thiol and amine groups was observed in the bands centered at 212 nm and 232 nm, associated with the nonbonding electron pairs from the amine and thiol groups. The *π*→π* transitions were also observed at 232 nm. In addition, the *n*→σ* transition of -C=O was observed at 290 nm. In the HYNIC-iPD-L1 spectrum (Figure 7), a redshift from 212 nm to 218 nm and the redshifts observed at 246 nm and 298 nm confirmed the chemical conjugation of iPDL1 to HYNIC. In addition, the absorption band at 246 nm also provides evidence for the modification of the -C-S bond in the conformation of the new HYNIC-iPD-L1 chemical structure. Similarly, the bathochromic shift from 225 nm and 283 nm (WL12 spectrum) to 229 nm and 288 nm (HYNIC-WL12), respectively, was indicative of the HYNIC and WL12 conjugation (Figure 7). 

### 3.3. Radiolabeling

**Figure 8:**
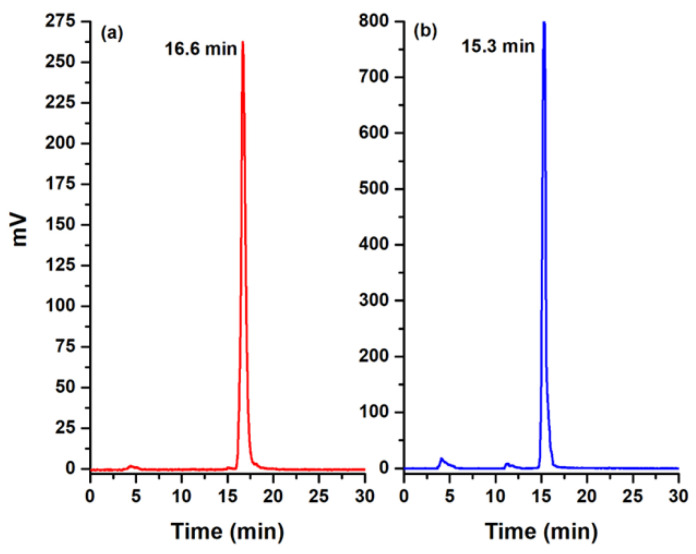
Radio-chromatogram of (**a**) [^99m^Tc]Tc-iPD-L1 and (**b**) [^99m^Tc]Tc-WL12. Reversed-phase column (C18, 3.9 × 300 mm, 10µm particle size).

The radiochemical purities of [^99m^Tc]Tc-iPD-L1 and [^99m^Tc]Tc-WL12 were 95.5 ± 1.5% without the need for additional purification, as determined by reversed-phase radio-HPLC (Figure 8). As expected, the retention time of [^99m^Tc]Tc-iPD-L1 was higher than that of [^99m^Tc]Tc-WL12, which is associated with their CLogP values of 6.38 and 5.78, respectively. Therefore, [^99m^Tc]Tc-iPD-L1 was found to be a more lipophilic compound compared to [^99m^Tc]Tc-WL12.

### 3.4. Stability

[^99m^Tc]Tc-iPD-L1 and [^99m^Tc]Tc-WL12 incubated in human serum at 37 °C for 30 min, 3 h, and 24 h remained stable. The chromatograms obtained with the radiometric detector and the UV–vis detector showed a retention time of the proteins between 4 and 7 min and a retention time for [^99m^Tc]Tc-iPD-L1, [^99m^Tc]Tc-WL12, and ^99m^TcO_4^−^_ of 9.8 min, 9.3 min, and 11.7 min, respectively. The radioactivity from [^99m^Tc]Tc-iPD-L1 bound to serum proteins was 1.3 ± 0.8% at 30 min, 3.2 ± 1.3% at 3 h, and 7.6 ± 1.9% at 24 h. High radiochemical stability (>90% at 24 h) was also observed. In the case of [^99m^Tc]Tc-WL12, protein binding was 1.0 ± 0.4% at 30 min, 3.3 ± 0.9% at 3 h, and 6.0 ± 1.3% at 24 h, with a radiochemical purity of >90% at 24 h. Therefore, there was no significant difference (*p* < 0.05, Student’s *t*-test) in protein binding and serum stability between the radiotracers.

### 3.5. Molecular Recognition by the PD-L1 Protein: Radio-SDS-PAGE

**Figure 9:**
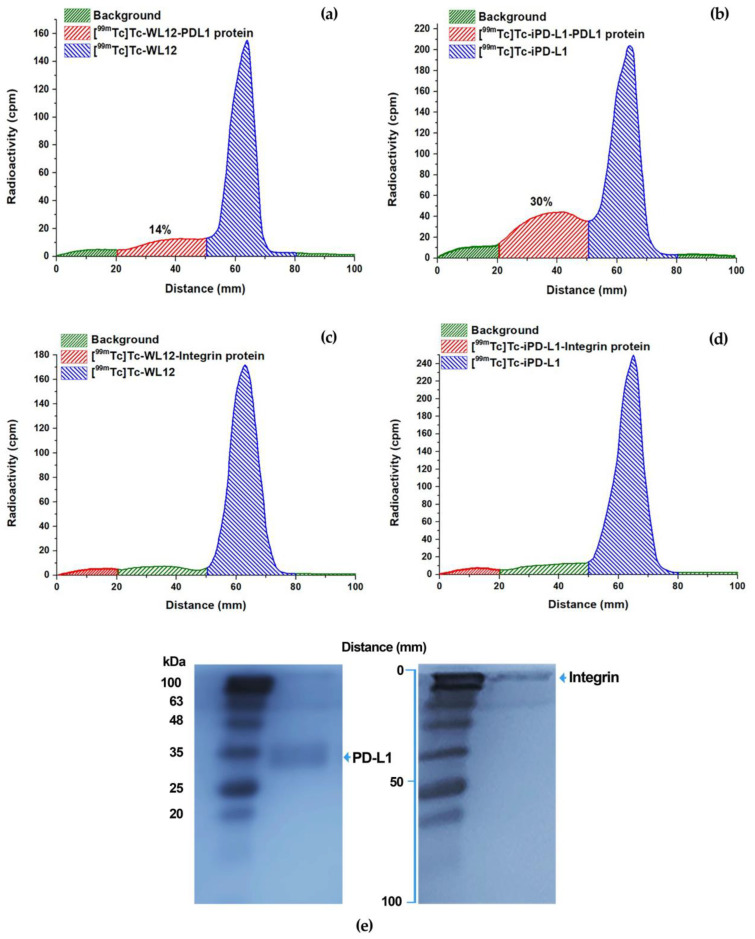
SDS-PAGE (radio-SDS-PAGE). Migration of [^99m^Tc]Tc-iPD-L1 (Rf = 0.6–0.7; 60–70 mm) and [^99m^Tc]Tc-WL12 (Rf = 0.6–0.7; 60–70 mm). (**a**) Interaction [^99m^Tc]Tc-WL12-PDL1 protein showing 14% of the radioactivity shifted at a distance associated with the PD-L1 protein. (**b**) Interaction [^99m^Tc]Tc-iPD-L1-PDL1 protein showing 30% of the radioactivity shifted at a distance associated with the PD-L1 protein. (**c**,**d**) No shift in radioactivity when [^99m^Tc]Tc-WL12-PDL1 and [^99m^Tc]Tc-iPD-L1-PDL1 interact with the integrin protein (negative control). (**e**) Migration of PD-L1 (Rf = 0.4; 40 mm) and integrin (Rf = 0.0; 0.0 mm) in the gel plate.

In vitro-specific recognition of [^99m^Tc]Tc-iPD-L1 and [^99m^Tc]Tc-WL12 by the PD-L1 protein was performed using a combination of radio-TLC and SDS-PAGE (radio-SDS-PAGE). After running samples on the gel by electrophoresis (total distance 100 mm), pure samples migrated in the plate as follows: PD-L1 (Rf = 0.4; 40 mm), integrin (Rf = 0.0; 0.0 mm), [^99m^Tc]Tc-iPD-L1 (Rf = 0.6–0.7; 60–70 mm), and [^99m^Tc]Tc-WL12 (Rf = 0.6–0.7; 60–70 mm). Therefore, the radioactivity associated with free peptides is in the front of the gel plate (around 60–70 mm). However, when the PD-L1 protein recognizes radiopeptides, the radioactivity is shifted to the distance associated with the protein migration (nearly 40 mm). As can be observed in Figure 9, when the interaction [^99m^Tc]Tc-WL12-PDL1 protein occurred, 14% of the radioactivity was detected at a distance associated with the PD-L1 protein (about 40 mm) (Figure 9a), indicating specific molecular recognition, since there was no shift in radioactivity when [^99m^Tc]Tc-WL12-PDL1 interacted with the control protein (integrin) (Figure 9c). In the case of the [^99m^Tc]Tc-iPD-L1 radiopeptide, the percentage of radioactivity associated with protein recognition was 30% (Figure 9b), with no shift in radioactivity when interacting with the integrin protein (negative control) (Figure 9d). Of course, a 100% shift in radioactivity is not expected because the number of moles of PDL1 and WL12 exceeds that of the protein. Nevertheless, as shown in Figure 9, the radio-SDS-PAGE assay can discriminate whether a small molecule radioligand is recognized by a specific protein or not. However, to confirm this specificity, the uptake and internalization of [^99m^Tc]Tc-iPD-L1 and [^99m^Tc]Tc-WL12 were also evaluated in cells expressing the PD-L1 protein, as confirmed by immunofluorescence as described below.

### 3.6. Specific Cellular Uptake and Internalization

**Figure 10:**
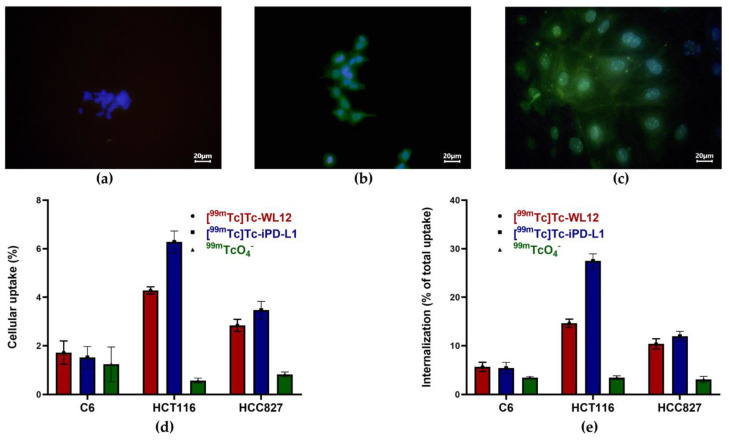
Micrographs (40x) of immunofluorescence staining for PD-L1 in (**a**) C6 cells, demonstrating negative PD-L1 expression, (**b**) HC116 cells (PD-L1 positive), and (**c**) HCC827 cells (PD-L1 positive); (merged images: cell nuclei stained in blue with DAPI and PD-L1 stained in green with anti-PD-L1). (**d**) Cellular uptake and (**e**) internalization of [^99m^Tc]Tc-WL12, [^99m^Tc]Tc-iPD-L1, and ^99m^TcO_4^−^_in C6, HCT116, and HCC827 cells. Two-way ANOVA statistical analysis (alpha 0.05) showed that there were significant differences (*p* < 0.0001) in the percentage of uptake and internalization due to both the cell type factor (C6, HCT116, and HCC827) and the radiotracer type ([^99m^Tc]Tc-WL12, [^99m^Tc]Tc-iPD-L1, and ^99m^TcO_4^−^_).

Figure 10 shows the micrographs of immunofluorescence staining for PD-L1 in C6 cells showing negative PD-L1 expression (Figure 10a) and positive expression for HC116 colorectal cancer cells and HCC827 lung adenocarcinoma cells (Figure 10b,c). Therefore, the significantly higher uptake and internalization of [^99m^Tc]Tc-WL12 and [^99m^Tc]Tc-iPD-L1 in HCT116 and HCC827 cells compared to C6 cells is due to specific recognition by the PD-L1 protein (Figure 10d,e). This behavior was also supported by the low ^99m^TcO_4^−^_ (negative control) uptake observed in all cells. A two-way ANOVA statistical analysis (alpha 0.05) showed that there were significant differences (*p* < 0.0001) in the percentage of uptake and internalization due to both the cell type factor (C6, HCT116, and HCC827) and the radiotracer type ([^99m^Tc]Tc-WL12, [^99m^Tc]Tc-iPD-L1, and ^99m^TcO_4^−^_).

### 3.7. Biodistribution

**Figure 11:**
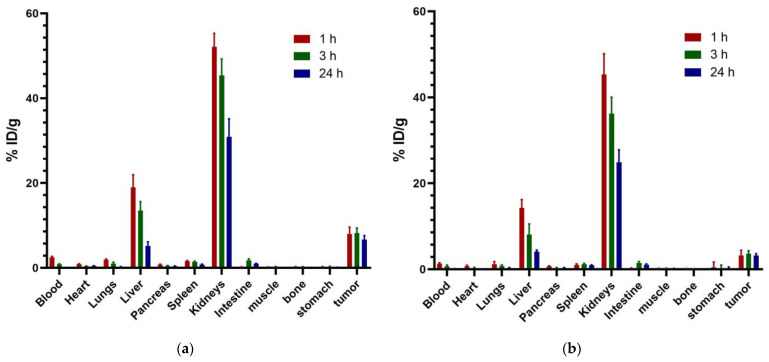
(**a**) Biodistribution of [^99m^Tc]Tc-iPD-L1 in mice bearing HCC827 lung cancer tumors at 1, 3, and 24 h after administration. (**b**) Biodistribution of [^99m^Tc]Tc-WL12 in mice bearing HCC827 lung cancer tumors at 1, 3, and 24 h after administration.

The evaluation of the biodistribution of [^99m^Tc]Tc-WL12 and [^99m^Tc]Tc-iPD-L1 in nude mice with induced lung cancer tumors (HCC827 cells) showed a tumor uptake of 3.22 ± 1.20% and 6.98 ± 0.89% of the injected dose/g, respectively, at 1 h after injection (Figure 11). After 24 h, the activity in tumors was 3.21 ± 0.45 %ID/g ([^99m^Tc]Tc-WL12) and 5.65 ± 0.98 %ID/g ([^99m^Tc]Tc-iPD-L1). Two-way ANOVA statistical analysis (alpha 0.05) showed that there were significant differences (*p* < 0.0001) in the percentage of tumor uptake over time due to the radiotracer type as follows: mean of [^99m^Tc]Tc-WL12 = 6.617; mean of [^99m^Tc]Tc-iPD-L1 = 3.347; difference between means: 3.270; SE of difference = 0.4414; 95% CI of difference = 2.308 to 4.232. Both radiopharmaceuticals showed hepatobiliary and renal elimination. Although [^99m^Tc]Tc-iPD-L1 demonstrated a significantly higher tumor uptake compared to [^99m^Tc]Tc-WL12, higher activity in blood, liver, and kidney was also observed for the ^99m^Tc-labeled iPD-L1 peptide. This behavior is related to the higher lipophilicity of [^99m^Tc]Tc-iPD-L1 compared to [^99m^Tc]Tc-WL12. Nevertheless, both radiotracers showed potential for the in vivo detection of PD-L1 expression in tumor lesions. 

### 3.8. Clinical Imaging

**Figure 12:**
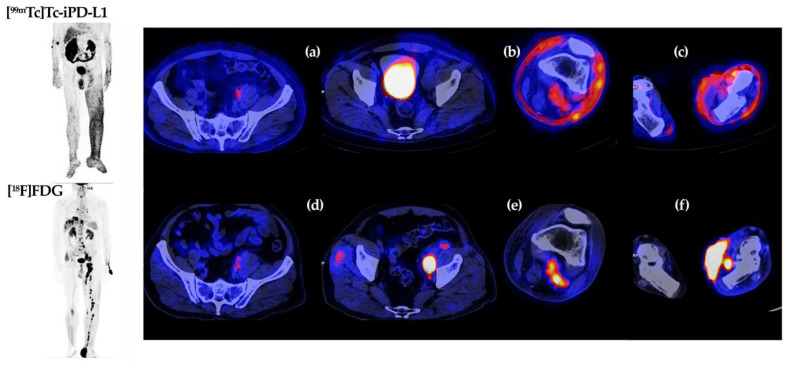
Molecular images of [^99m^Tc]Tc-iPD-L1 (top) and [^18^F]FDG (bottom) in a patient with plantar malignant melanoma. Axial plane at pelvic level: (**a**,**d**). Axial view of left lower leg: (**b**,**e**). Axial view of left foot (plantar lesion) and right foot: (**c**,**f**).

Figure 12 shows [^99m^Tc]Tc-iPD-L1 SPECT/CT imaging of tumor lesions and adenopathies expressing PD-L1 and [^18^F]FDG/PET imaging of lesions and adenopathies with high metabolic activity in a patient with plantar malignant melanoma. Table 2 provides a detailed analysis of the molecular imaging findings obtained in the [^99m^Tc]Tc-iPD-L1 SPECT/CT and [^18^F]FDG/PET studies. 

An interesting point in the image analysis is the uptake of [^99m^Tc]Tc-iPD-L1 in soft tissues, which could be related to PD-L1 expression in T cells due to the strong inflammatory response. Similarly, the knowledge of tumor heterogeneity of PD-L1 expression is strengthened by the absence of [^99m^Tc]Tc-iPD-L1 uptake in external pelvic iliac lymph nodes and the positive uptake in internal iliac lymph nodes.

## 4. Discussion

Docking studies are used in various phases of drug design, especially in predicting the docked conformation of a ligand–receptor complex and in classifying ligand molecules according to their binding energy [17]. In this study, the lowest energy was established as the main criterion for predicting the potential affinity of the iPD-L1 ligand compared to the WL12 peptide (positive control), whose affinity for the PD-L1 protein has been clearly established previously [8,18]. It is important to note the interactions of the lysine side chain (hydrazine-piridine: HYNIC) in the macrocycle of the tetradecapeptide, where the protonated molecule (-NH-NH_3_^+^) generates a simultaneous double interaction (ionic interaction/salt bridge and hydrogen bond) between the carboxylate ion (-COO^−^) of Glu-58 in the PD-L1 protein and the ammonium ion (-NH_3_^+^) of the hydrazine in iPD-L1 (Figure 3). It is well known that the stabilization energy due to ionic interactions (salt bridges) is many times higher than that of conventional hydrogen bonding interactions, but they can be added to increase the stabilization energy in a simultaneous interaction such as the one generated in the iPD-L1 molecule (Figure 3). This interface contributed to obtaining an anchoring structure with sufficient recognition by the PD-L1 ligand, without the need to add methylation sites in the amino acids, nor -S- bonds in the cyclic chain, as in the WL12 structure [8,18]. 

The potential of the novel Tc-99m-labeled iPD-L1 ligand for the in vivo detection of PD-L1 overexpression was demonstrated both in vitro and in vivo. However, it was shown that [^99m^Tc]Tc-WL12 may also be useful for the SPECT imaging of PD-L1. Although it showed lower tumor uptake, its less lipophilic nature compared to [^99m^Tc]Tc-iPD-L1 may allow images with better tumor lesion/background ratios. 

As an important point, it would be interesting to perform a clinical study comparing the biokinetics and affinity for PD-L1-positive tumors between the radiopharmaceuticals for SPECT so far reported, such as [^99m^Tc]Tc-iPD-L1, [^99m^Tc]Tc-WL12, and ^99m^Tc-labeled anti-PD-L1-single-domain antibodies [19,20,21], which would allow the establishing of the advantages and disadvantages between the use of inhibitory peptides and anti-PD-L1 radiotracers. However, a longer residence time in the circulating blood (slow clearance) and a longer time for tumor uptake and visualization of PD-L1-positive tumors (e.g., tumor visualization at 4 h post-injection with maximum uptake at 24 h [20]) are expected for the ^99m^Tc-anti-PD-L1 radiopharmaceuticals (complete antibodies) [20] compared to the ^99m^Tc-peptides examined in this study (tumor visualization at 1 h post-injection). The difference is due to the tumor/blood activity ratio <1 during the first hours after injection of the radiolabeled antibodies, whereas a tumor/blood ratio >5 is observed for the radiopeptides at 1 h after administration. On the other hand, the biokinetic behavior between ^99m^Tc-single domain antibodies and ^99m^Tc-peptides is expected to be quite similar [19,21].

Nevertheless, the most important aspect is the potential use that this type of radiopharmaceuticals is expected to have in clinical practice, where they will undoubtedly provide relevant information related to the phenotype of the tumor microenvironment in a personalized manner.

Blockade of the PD-1/PD-L1 checkpoint by immunotherapeutic techniques is a major advance in cancer treatment. However, it remains difficult to identify patients who are likely to benefit from this therapy because immunohistochemistry results cannot provide information on the heterogeneity of PD-L1 expression in all tumor lesions of a patient due to the invasive limitations of the technique. SPECT imaging is non-invasive and is the best option for patient selection and therapeutic follow-up in immunotherapy, as it is able to assess PD-L1 overexpression in whole-body scans. As demonstrated in this research, the use of [^99m^Tc]Tc-iPD-L1/SPECT in the patient with plantar malignant melanoma allowed the differentiation between lesions that express PD-L1 protein and those that do not, helping to determine whether or not immunotherapy is a good option for the patient’s treatment.

## 5. Conclusions

[^99m^Tc]Tc-iPD-L1 is a novel peptide radiotracer for SPECT imaging with a high affinity for the PD-L1 protein. In vivo evaluation demonstrated its ability to differentiate tumor lesions expressing PD-L1 protein from those that do not, making [^99m^Tc]Tc-iPD-L1 a potentially useful tool for guiding treatment decisions with anti-PD-L1 immunotherapy in cancer patients. The results obtained in this study warrant further dosimetric and clinical studies to determine the sensitivity and specificity of [^99m^Tc]Tc-iPD-L1/SPECT for PD-L1 expression imaging.

## Figures and Tables

**Table 1:** Scoring results and binding sites to PD-L1 protein of ligands iPD-L1, HYNIC-iPD-L1, WL12, and HYNIC-WL12.

Inhibitor Peptide	Affinity (kcal/mol)	Inhibition Constant (µM)	Binding Site
iPD-L1	−6.7	12.26	Lys25, Lys41, Leu94, Leu27, Phe42, Val23. Glu39, Lys124, Pro24, Thr22, Val44
HYNIC-iPD-L1	−7.2	5.27	Gln107, Arg125, Ala132, Leu106, Val111, Ile126, Glu60, Ala109, Met5, Thr127, Arg113, Lys129, Tyr112
WL12	−5.1	82.51	Ser34, Glu39, Glu31, Asn35
HYNIC-WL12	−6.0	39.95	Leu94, Leu88, Glu31, Gln100, Thr102, Ser34

**Table 2:** A molecular imaging study in a patient with malignant plantar melanoma using [^18^F]FDG and [^99m^Tc]Tc-PD-L1. Lesions and adenopathies positive for high metabolic activity were detected with [^18^F]FDG (✓). Diagnostic impression of [^99m^Tc]Tc-PD-L1 study showed lesions and adenopathies with (†) and without (×) PD-L1 overexpression.

Adenopathy/Lesion	[^18^F]FDG Uptake	[^99m^Tc]Tc-iPD-L1 Uptake
Mediastinal adenopathies level 4R, 8 and 10L up to 12 mm	✓	×
Left retrocrural adenopathy of 9 mm	✓	×
Liver with heterogeneous appearance due to the presence of poorly defined hypodense images distributed in an allotropic manner, the largest in segment 7	✓	×
Retroperitoneal paraaortic and intercavoaortic adenopathies of 14 mm	✓	×
Adenopathies of the common, internal, external, and obturator iliac chains of left predominance up to 26 mm	✓	×
Left inguinal adenopathies forming clusters of 29 mm	✓	†
Left femoral adenopathies of 12 mm	✓	†
Left popliteal hollow adenopathies up to 20 mm	✓	†
Adenopathies in the entire intramuscular tract of the left lower extremity up to 14 mm	✓	†
A lesion with an exophytic component with poorly defined oval edges with heterogeneous soft tissue density due to the presence of air corpuscles in its interior, located in the heel region with extension to the medial region of the left foot measuring 67 × 35 mm	✓	†

## Data Availability

Data are contained within the article.
